# Anthropogenic noise and light alter temporal but not spatial breeding behavior in a wild frog

**DOI:** 10.1093/beheco/arac077

**Published:** 2022-08-20

**Authors:** Andrew D Cronin, Judith A H Smit, Wouter Halfwerk

**Affiliations:** Amsterdam Institute for Life and Environment, Vrije Universiteit, De Boelelaan, HV, Amsterdam, The Netherlands; Amsterdam Institute for Life and Environment, Vrije Universiteit, De Boelelaan, HV, Amsterdam, The Netherlands; Amsterdam Institute for Life and Environment, Vrije Universiteit, De Boelelaan, HV, Amsterdam, The Netherlands

**Keywords:** *anthropogenic noise*, *artificial light at night*, *breeding behavior*, *multisensory pollution*, *sexual communication*, *urbanization*

## Abstract

Increasing urbanization has led to large-scale land-use changes, exposing persistent populations to drastically altered environments. Sensory pollutants, including low-frequency anthropogenic noise and artificial light at night (ALAN), are typically associated with urban environments and known to impact animal populations in a variety of ways. Both ALAN and anthropogenic noise can alter behavioral and physiological processes important for survival and reproduction, including communication and circadian rhythms. Although noise and light pollution typically co-occur in urbanized areas, few studies have addressed their combined impact on species’ behavior. Here, we assessed how anthropogenic noise and ALAN can influence spatial and temporal variation in breeding activity of a wild frog population. By exposing artificial breeding sites inside a tropical rainforest to multiple sensory environments, we found that both anthropogenic noise and ALAN impact breeding behavior of túngara frogs (*Engystomops pustulosus*), albeit in different ways. Males arrived later in the night at their breeding sites in response to anthropogenic noise. ALAN, on the other hand, led to an increase in calling effort. We found no evidence that noise or light pollution either attracted frogs to or repelled frogs from breeding sites. Thus, anthropogenic noise may negatively affect calling males by shifting the timing of sexual signaling. Conversely, ALAN may increase the attractiveness of calling males. These changes in breeding behavior highlight the complex ways that urban multisensory pollution can influence behavior and suggest that such changes may have important ecological implications for the wildlife that are becoming increasingly exposed to urban multisensory pollution.

## Teaser

Urbanization exposes animal populations to sensory pollutants, including anthropogenic noise and artificial light, possibly affecting survival and reproductive success. We examined the effects of noise and light pollution on breeding behavior in a wild population of frogs. Frogs called later in the night with anthropogenic noise, potentially increasing predation and reducing reproductive success. Light increased the amount of frog calling, suggesting that light might play an important role in altering communication in urban frog populations.

## INTRODUCTION

Urbanization has led to the rapid transformation of land across the globe, and the expansion of urban areas is expected to continue (United Nations 2018). These land-use changes will likely continue to cause local decreases in biodiversity for many taxa (McKinney 2008; Seto et al. 2012). The species able to persist in these highly modified urban environments must nevertheless face new environmental stressors, including evolutionarily novel pressures, such as low-frequency anthropogenic noise (Shannon et al. 2016) and artificial light at night (ALAN) (Kyba et al. 2015). Many animals in urban areas must therefore adapt to these sensory pollutants, on ecological and/or evolutionary timescales, or face the risk of going locally extinct (Swaddle et al. 2015; Hopkins et al. 2018).

The breeding behavior and sexual communication of many species has been shown to be influenced by sensory pollutants (Candolin and Wong 2019; Heinen-Kay et al. 2021; Cronin et al. 2022). These sensory pollutants can change breeding site use (Francis et al. 2011), alter timing of breeding activity (Kempenaers et al. 2010; Halfwerk et al. 2011), or influence sexual signaling (Parris et al. 2009; Van Geffen et al. 2015; Halfwerk et al. 2019; Elgert et al. 2020). For example, the reduction in urban noise during the COVID-19 pandemic enabled researchers to show the strong effects of urban noise pollution on several song characteristics, including by reducing minimum trill frequency, in the white-crowned sparrow *(Zonotrichia leucophrys*) (Derryberry et al. 2020). Most work, however, has focused on the isolated effects of sensory pollutants, although the combined effects of multiple anthropogenic stimuli has recently garnered more attention (Halfwerk and Slabbekoorn 2015; McMahon et al. 2017; Ferraro et al. 2020; Willems et al. 2021; for review of the combined effects of anthropogenic noise and ALAN, see Halfwerk and Jerem 2021). As sensory pollutants typically covary in urban environments, single exposure studies may fail to fully demonstrate the impacts of urban-associated stimuli on breeding behaviors, potentially missing pronounced population level consequences (Candolin and Wong 2019; Cronin et al. 2022).

Sexual communication can directly be influenced by sensory pollutants, either resulting in changes in signal production or signal perception (reviewed in Cronin et al. 2022). For example, noise and light pollution can both influence the composition and intensity of sexual signals (Van Geffen et al. 2015; Halfwerk et al. 2016; Elgert et al. 2020). Spatial variation in breeding activity is also likely to be influenced by both anthropogenic noise and ALAN. Previous work has, e.g., demonstrated patterns of attraction or repulsion to areas with anthropogenic noise and ALAN in several species across different taxa (Bayne et al. 2008; Francis et al. 2011; Russ et al. 2017; Hennigar et al. 2019; Parkinson et al. 2020; Kosicki 2021; Wilson et al. 2021), but the specific role that ALAN and anthropogenic noise play remains unclear. Attraction and repulsion to certain sites may also impact sexual signaling indirectly, as many species advertise their sexual signals from specific locations that coincide with optimal conditions for communication. Frogs, for example, advertise their readiness to mate from specific calling sites that best match their signal design (Muñoz et al. 2020).

Anthropogenic noise and artificial light at night are also known to influence the timing of breeding activity, especially for the nocturnal or crepuscular species. For example, both ALAN (Da Silva et al. 2014) and airplane noise (Alquezar et al. 2020) advanced the timing of dawn singing in various bird species. Such temporal shifts in breeding activity may have detrimental effects, as the timing and duration of breeding activity can be under strong sexual and natural selection and often coincides with the optimal period or “window of opportunity” to produce breeding displays (Grant et al. 2013). These “windows of opportunity” may arise due to a variety of different factors, including utilizing optimal ambient conditions for signal transmission (Wiley and Richards 1978; Henwood and Fabrick 1979), predatory satiation (Sweeney and Vannote 1982), timing of prey availability (Berg et al. 2006), and predator avoidance (Morgan and Christy 1995). Regardless of the precise mechanisms at play, breeding activity within these “windows of opportunity” is likely to confer some adaptive benefits. Despite many studies documenting temporal shifts in communication in relation to anthropogenic sensory pollution (Arroyo-Solís et al. 2013; Nordt and Klenke 2013; Silva et al. 2014; Dias et al. 2019; Alquezar et al. 2020), the potential fitness consequences of these shifts are often unclear, due to a lack of information on the costs and benefits.

We studied the combined impacts of anthropogenic noise and ALAN on breeding behavior and sexual signaling in a wild population of túngara frog, *Engystomops pustulosus*. This species is readily found in natural as well as anthropogenically disturbed areas characterized by high levels of sensory pollution (Halfwerk et al. 2019). Males of this species produce mating calls to attract females, but can also attract a variety of eavesdroppers including predatory bats and parasitic midges (Ryan et al. 1982; Bernal et al. 2006; McMahon et al. 2017). Males have a multicomponent call, consisting of a frequency modulated whine followed by the voluntary addition of harmonically rich “chucks”. Adding chucks increases the complexity and relative attractiveness of a male’s call (Ryan 1985). Like many frog species, túngara frogs often call in choruses, and call primarily in the first part of the night (Ryan 1983). This relatively narrow window of breeding activity is likely because of a balance of maximizing attraction of intended receivers (females), while minimizing the attraction of unintended receivers (including predatory bats). Shifts in the timing of male signaling in response to sensory pollutants could therefore have significant consequences on the likelihood of mate attraction and predation.

We studied the túngara frog’s breeding behavior at artificial breeding sites placed in the field while manipulating light and noise stimuli following a full-factorial experimental design. We specifically tested if isolated versus combined anthropogenic noise and ALAN-altered breeding behavior at three levels: 1) breeding site use, 2) timing of breeding activity, and 3) male signaling traits. In addition, we recorded receiver activity, including mate attraction and predator attraction, to examine any potential fitness consequences.

## METHODS

### General Methods

All experiments were performed at the Smithsonian Tropical Research Institute (STRI) facilities on Barro Colorado Island, Panamá, between July and August 2019. All experiments were performed under the approval of STRI (IACUC permit: 2019-0301-2022) and Autoridad Nacional del Ambiente de Panamá (SE/A-47–19). We placed 32 artificial breeding sites, which were plant dishes (Ø 50 cm), in the forest that were at least 10 m from the nearest trail to reduce any human-related disturbances and > 75 m from each other. Additionally, no calling activity was heard from areas surrounding the artificial breeding sites, indicating that breeding sites were isolated from any natural choruses. As this species requires water to call and to breed, we filled these artificial breeding sites with water collected from another active breeding site. All the artificial breeding sites were filled with water from the same source. After a total of 32 breeding sites were placed in the field, we conducted nightly surveys of these sites and noted any túngara frog activity (calling, individuals within the puddle, or foam nests). Once frog activity was documented, which occurred in 19 breeding sites, these sites were monitored either with video (Reconyx Ultrafire RX9 Trail Camera) or audio recorders (Wildlife Acoustics SM4 Songmeter). Frogs occupied breeding sites an average of 12.11 days after they were placed in the field.

After four artificial breeding sites had become active, we installed setups to broadcast noise and light stimuli at each site and monitored these sites with our video recorders. One of four treatments was then presented to a puddle on a given night: Control (C), Light (L), Noise (N), and Light + Noise (L + N). Treatments were randomized and balanced within a block, ensuring that all the treatments were presented at sites within a single block on the same night. Treatments were changed each day at each site, so that over the course of a round, all sites in a block received all treatments. We included 16 of the 19 sites with breeding activity in our experiments. Experiments lasted between 18:00 and 23:00, starting approximately 40 minutes before sunset, culminating in 533.5 hours of video and audio data. Each block of sites received either one round (4 days) or two rounds (8 days) of treatment exposure. In one block, equipment failure caused us to repeat a night of trials.

The ALAN and anthropogenic noise stimuli were placed 3 meters away from the puddle, and the video camera was set up opportunistically around the puddle (between 0.5 and 2 meters). We simulated urban anthropogenic noise by recording urban noise from four sites surrounding Panama City, Panama. We then selected two 5-minute segments from each site. In order to create a standardized, but still ecologically relevant noise stimuli, we synthesized “urban white noise”. This was accomplished with R packages *seewave* (version 2.1.3) (Sueur et al. 2008) and *tuneR* (version 1.3.2) (Ligges et al. 2018) by analyzing the spectra of urban noise recordings, and subsequently shaping noise spectra to match the urban recordings. We then manually adjusted amplitude profiles to mimic the amplitude modulations on the recording. These stimuli were used to create a 40-minute loop, which was repeated for the duration of the experiment. We measured the minimum and maximum amplitudes of the urban noise stimuli in the field with an SPL meter (Voltcraft SL-100, A-weighted, fast window), and recorded a range of 49.4–58.8 dB measured at the breeding site. ALAN was generated by a headlamp (Black Diamond SpotLite) with a diffusing filter to create an even spread of light, creating an average light level of 1.53 lux at the breeding site (range: 1.06–3.1 lux), measured by a lux meter (HT Instruments HT309). These noise and light levels lie within the range experienced by urban populations (Halfwerk et al. 2019).

### Video analysis

Videos were scored primarily by one experimenter (A.D.C.). The scorer was blind to the sensory treatment except for a small subset of videos in which frogs were out of view, in which case the audio file was checked for calling behavior. We scored breeding site use as the presence/absence of frogs within the breeding site for each recording period. Each recording period was a 10-minute interval, resulting in a maximum of 30 recording periods per night per site. Additionally, we counted the maximum number of frogs in the puddle at a given time. For the presence of calling, we scored the presence/absence of any calling within a recording period, along with the total number of calling frogs. If a frog left the view within a recording period and another frog entered, it was assumed to be the same frog unless there was strong evidence to assume it was a new individual (i.e., differing exit/entrance angles, timing between exit and entrance, or the presence of distinctive traits, such as a missing limb). Mating behaviors (such as amplexus or nest building) were also scored, in addition to any predation attempts on the frogs. For the timing of breeding activity, we documented two distinct measures. Breeding site arrival time was calculated as the number of minutes from the start of the experiment (18:00 h) until the first adult frog entered the puddle. Calling start time was measured as the difference between when the first frog started calling and when the frog arrived at the breeding site. This measure was therefore independent of arrival time at the breeding site. In two instances, frogs were already present and calling when the experimental period began, and these data points for both breeding site arrival and calling start time were removed.

### Call Analysis

For call analyses, we randomly selected and scored five recording periods in which calling was present per night per site. Call analysis was conducted by one experimenter (A.D.C.) and J. Mariën. If five or fewer recordings were available for a site on a given night, we analyzed all period. Because there were limited instances when more than one male called, and male calling behavior is highly influenced by calling competitors (Bernal et al. 2009), we limited our analyses to periods when a single male was calling. We defined calling bouts as the occurrence of at least 10 calls with no more than 10 seconds between each call. A calling bout would end when there was more than 10 seconds between two calls. Although arbitrary, we found that 10 seconds functioned as a cutoff point for a calling bout, as pauses longer than 10 seconds were often much longer. We use the likelihood of producing a calling bout as a proxy for calling effort. After identifying calling bouts, we further analyzed the first calling bout in each 10-minute recording. For each calling bout, we started our analysis 15 seconds after the first call in order to include a “warm-up” period. After these 15 seconds, we scored the call rate (calls/minute), average call complexity (chucks/call), and maximum complexity (most chucks in a single call) for 60 seconds, measures known to be important in mate and predator attraction (Rand and Ryan 1981; Ryan et al. 1982).

### Data Analysis and Statistics

We conducted all the statistical analyses using R version 3.5.3 (R Core Team 2019) on the RStudio interface (RStudio Team 2016), and plots were generated using *ggplot2* (version 3.3.5) (Wickham 2016). Models were created and analyzed using either the *lme4* package (version 1.1.27.1) (Bates et al. 2015) or the *glmmTMB* package (version 1.1.3) (Brooks et al. 2017). Because of our interest in both the additive and interactive effects of light and noise, as well as the full-factorial experimental design, all models incorporated light and noise and their interaction as fixed factors. Presence/absence data (breeding presence, call presence, and calling effort) were analyzed using a binomial generalized linear mixed model with a logit link function. For both breeding site arrival time and calling start time, we utilized Poisson family distribution models, due to the distribution and zero-bounded nature of these data. Additionally, because of under-dispersion, we used a generalized Poisson model (genpois) with a log link function for breeding site arrival time. Calling characteristics (aside from calling effort) were modeled with a linear mixed model using the identity link function. Because of the males producing a very limited number of chucks, we were unable to run any analyses on call complexity. We began by assessing optimal structure of null models for each dependent variable separately, based on the lowest AIC values. For all models, we tested different random structures, including date, site, and days active (the number of days since the breeding site was placed in the field). For calling traits, we also tested the time of night as a random effect, but none of the final null models contained this factor. For all models, the best random structure included date nested within site. Additionally, for breeding site presence and calling presence, the number of days active was included as a random intercept. We visually inspected normality of residuals using QQ plots of residuals and checked for heteroskedasticity by plotting residuals and fitted values when appropriate. All binomial and the Poisson family models were checked for over- and under-dispersion and zero inflation with the *dHARMA* package (version 0.4.4) (Hartig 2021). Call bout length was log-transformed in order to meet model assumptions.

For model comparisons, we utilized a backward selection approach, and evaluated the influence of our fixed effects with likelihood ratio tests. For significant interactions, we examined if a combination of light and noise acted synergistically or antagonistically by comparing interaction estimates with the expected additive effects of light and noise (Hale et al. 2017). When there was a significant interaction, model estimates for each fixed effect were taken from the full model. If there was no significant interaction, model estimates for fixed effects were taken from the reduced model. We have set the statistical significance levels to *p* < 0.05, and additionally report all trends (*p* < 0.1).

## RESULTS

The number of frogs within the breeding site at a given moment ranged from 0 to 7, with an overall average of 0.43 frogs/recording period. In intervals where at least one frog was present, calling occurred 59% of the time, with several instances of two males calling at the same time (4%). There were 14 instances of amplexus, split almost evenly among treatments. We documented no predation attempts throughout the duration of this experiment. Due to the low occurrence of amplexus and the lack of predation attempts, we were unable to further analyze either of these responses to ALAN or anthropogenic noise.

### Breeding Site Use and Timing of Breeding Activity

**Figure 1:**
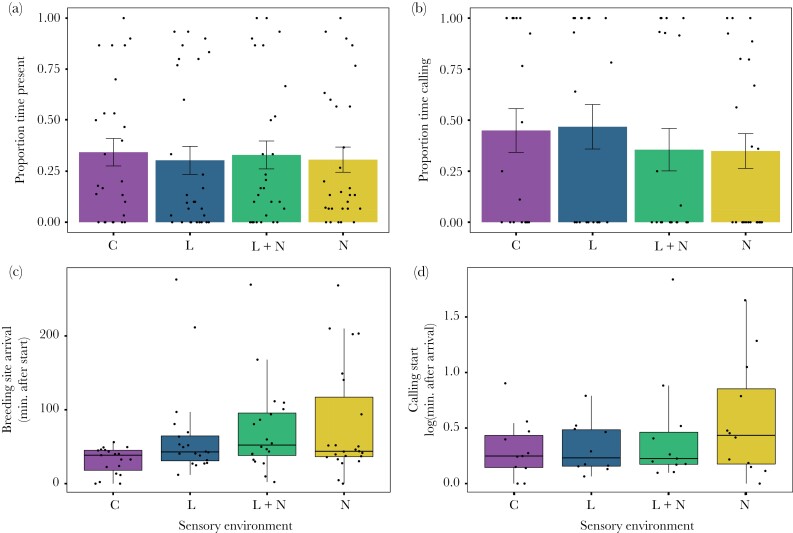
The effects of sensory pollutants on breeding site use (a-b), and timing of breeding activity (c-d). a) Breeding site presence (n= 3201 recording periods, from 16 sites and 22 nights) and b) presence of calling, are represented by a mean per site/per night (*n* = 877 recording periods, from 16 sites and 22 nights). c) breeding site arrival time (*n* = 82, from 16 sites and 22 nights), and d) calling start time (*n* = 44, from 10 sites and 18 nights). Means are presented for proportional data (a-b), and boxplots of raw data are presented in all other graphs (c-d). Error bars represent standard error. See Table 1 for model outputs and statistics.

Breeding site use was not affected by either ALAN or anthropogenic noise. We found no interaction between light and noise, and no isolated effect of light or noise for either frog presence at breeding sites or the presence of calling activity (all *p* > 0.18) (Fig. 1a-b, Table 1). For breeding site arrival time, we did find a significant interaction between noise and light (*n* = 82 observations from 16 sites, likelihood ratio test: ***χ***^2^ = 5.10, *p* = 0.02). The nature of this interaction was antagonistic, as the estimated additive effects of light and noise (+62 minutes) were 29 minutes higher than the observed interaction term (+33 minutes) (Table 1). Noise as an isolated effect also significantly altered breeding site arrival time (***χ***^2^ = 5.17, *p* = 0.02), delaying the arrival time relative to the control (estimated effect: +33 minutes). Light as an independent factor also showed a trend to delay breeding site arrival time (estimated effect: +29 minutes), although this was not statistically significant (***χ***^2^ = 2.79, *p* = 0.09) (Fig. 1c, Table 1). We found no interaction between noise and light for calling start time (*p* = 0.73). There was an additional trend showing a delay in call timing due to noise (estimated effect: +1 minute; ***χ***^2^ = 2.94, *p* = 0.08). We found no significant effect of light on calling start time (*p* = 0.41) (Fig. 1d, Table 1).

**Table 1:** Results of GLMM, testing the effects of light, noise, and their interaction on breeding site use, the presence of calling activity, and timing of breeding behaviors. Interaction estimates and standard error (SE) are derived from the full model. All other estimates and SE are derived from reduced models, with non-significant interaction terms removed. Estimates and standard error are not back transformed.

Response	Explanatory Variable	Estimate	SE	*χ2*	*P* value
*Breeding site presence*	Intercept	-1.95	0.6		
	Light*Noise	0.46	0.79	0.33	0.57
	Light	-0.18	0.4	0.21	0.65
	Noise	0.56	0.41	1.73	0.19
*Calling presence*	Intercept	-2.39	1.82		
	Light*Noise	0.17	2.18	0.01	0.94
	Light	0.06	1.08	0	1
	Noise	-1.12	1.11	1.03	0.31
*Breeding site arrival time*	Intercept	3.43	0.2		
	Light*Noise	-0.65	0.37	5.1	**0.02**
	Light	0.66	0.27	2.79	0.09
	Noise	0.72	0.27	5.17	**0.02**
*Calling start time*	Intercept	-0.27	0.51		
	Light*Noise	-0.39	1.12	0.12	0.73
	Light	-0.45	0.56	0.67	0.41
	Noise	0.98	0.56	2.94	0.08

### Signaling Traits

**Figure 2:**
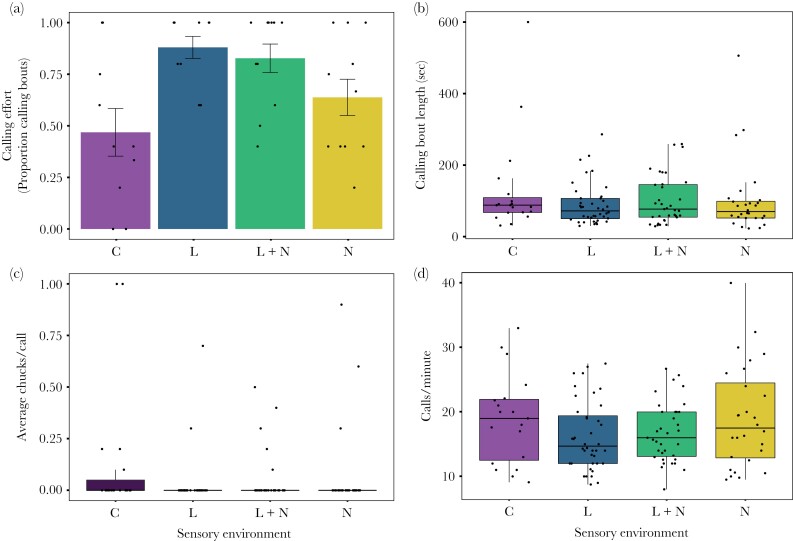
The effects of sensory pollutants on signaling traits. a) Calling effort (*n* = 186 recording periods, from 11 sites and 19 nights), b) calling bout length (n = 122 calling bouts, from 8 sites and 17 nights), c) call complexity (*n* = 122 calling bouts, from 8 sites and 17 nights), and d) call rate (*n* = 122 calling bouts, from 8 sites and 17 nights). Means are presented for proportional data (a), and boxplots of raw data are presented in all other graphs (b-d). Error bars represent standard error. See Table 2 and text for statistics.

We found no interaction between noise and light with respect to calling effort (*n* = 186 observations from 11 sites, ***χ***^2^ = 1.13, *p* = 0.29), and noise in isolation did not alter calling effort (***χ***^2^ = 0.06, *p* = 0.81). However, light did significantly increase calling effort (***χ***^2^ = 12.8, *p* < 0.001, log odds estimate: +1.82) (Fig. 2a, Table 2). Within calling bouts, we found no interaction or influence of either sensory pollutant on call bout length (all *p* > 0.17) (Fig. 2b, Table 2). Similarly, call rate did not differ in response to the isolated effects of either light or noise or their interaction (all *p* > 0.49) (Fig. 2d, Table 2). Complex calling occurred too infrequently to test the effect of light and noise, and was therefore not incorporated into any analyses.

**Table 2:** Results of GLMM, testing the effects of light, noise, and their interaction on sexual signaling traits. We were unable to analyze call complexity due to the rarity of complex calls. Interaction estimates and standard error (SE) are derived from the full model. All other estimates and SE are derived from reduced models, with non-significant terms removed. Estimates and standard error are not back transformed.

Response	Explanatory Variable	Estimate	SE	*χ2*	*P* value
*Calling effort*	Intercept	0.2	0.44		
	Light*Noise	-0.94	0.89	1.13	0.29
	Light	1.82	0.49	12.8	**<0.001**
	Noise	0.11	0.44	0.06	0.81
*Call bout length*	Intercept	1.93	0.06		
	Light*Noise	0.14	0.10	1.8	0.18
	Light	-0.01	0.05	0.03	0.86
	Noise	-0.03	0.05	0.31	0.58
*Call rate*	Intercept	17.91	1.63		
	Light*Noise	0.68	2.57	0.05	0.82
	Light	-0.77	1.34	0.42	0.52
	Noise	0.94	1.27	0.46	0.5

## DISCUSSION

Both ALAN and anthropogenic noise showed effects on the breeding behavior of túngara frogs, although each sensory pollutant altered different aspects of breeding activity and sexual signaling. Anthropogenic noise was found to delay when frogs arrived at breeding sites, whereas ALAN exposure increased calling effort. Due to low sample sizes, we were unable to assess the effects of ALAN or anthropogenic noise on mate or predator attraction.

Our study experimentally demonstrates that anthropogenic noise, and possibly ALAN, can alter the timing of breeding activity of anuran amphibians. Under control conditions, males always arrived within an hour after start of the experiment, whereas at noise polluted sites, males would on average arrive + 33 minutes later compared with the control, and sometimes up to three hours later. The presence of ALAN showed a similar trend, delaying the timing of breeding site arrival by + 29 minutes, although this was non-significant. In avian species, where most previous research has focused, the relative importance of specific sensory pollutants on the timing of breeding activity has shown mixed results and suggests that the effects of sensory pollutants on spatio-temporal breeding behaviors is highly species-specific (Kempenaers et al. 2010; Arroyo-Solís et al. 2013; Dias et al. 2019; Alquezar et al. 2020; Senzaki et al. 2020). Several studies have demonstrated a particularly large effect of ALAN on activity patterns (Kempenaers et al. 2010; Dominoni et al. 2013; Silva et al. 2014); however, other studies have suggested that anthropogenic noise may play a predominant role (Fuller et al. 2007; Arroyo-Solís et al. 2013; Nordt and Klenke 2013; Alquezar et al. 2020). In many cases, the relationships between the timing of sexual signaling and these two sensory pollutants remain unclear. This lack of clarity is likely due in part to the correlative nature of most previous studies, which are often conducted in natural populations experiencing varying degrees of light and noise pollution. While these studies provide a useful starting point and may give insight into how organisms deal with these sensory pollutants, attributing causality to ALAN and anthropogenic noise may be difficult without experimental manipulations. In our study, we found a clear role of anthropogenic noise in the timing of breeding activity, as well as a trend of a delaying effect of ALAN. Additionally, the combined effect of light and noise pollution was antagonistic in nature, likely due both to a “ceiling effect” in response to noise and the fact that the isolated effect of light was relatively large, but not statistically significant (see Halfwerk and Jerem 2021 for a discussion on the ceiling effect in the context of multisensory pollution). As with many dawn-singing avian species, the delay documented in this study may represent a significant shift for túngara frogs, as breeding activity in this species is confined to a relatively narrow time period.

Shifts in the timing of breeding activity have the potential to alter the efficacy of certain breeding behaviors, as well as the costs and benefits associated with sexual signaling. In the case of túngara frogs, the costs and benefits of delayed signaling can change via several mechanisms (Page et al. 2013). A delayed start of calling could lead to an increased risk of predation. One of the primary predators of túngara frogs, the predatory bat *Trachops cirrhosus*, eavesdrops on frog vocalizations to localize and capture calling male túngara frogs (Ryan et al. 1982). Although we found no predation attempts throughout the duration of our study, these predators exert a significant pressure on túngara frogs (Ryan et al. 1981). Previous tracking of *T. cirrhosus* near our field sites indicates that these bats usually leave roosts at and immediately following sunset, between 18:30 and 19:00 (Jones et al. 2017), whereas at our control puddles, males always arrived before 19:00. These findings suggest that earlier male calling may be related to a decrease in predation risk, as the predation risk will increase over time until all bats have left the roost and have traveled to túngara frog breeding sites (at or after 19:00). In addition to facing increased predation risk, earlier calling males may also have additional benefits conferred via mating success. In our dataset, we observed 14 instances of amplexus, with more than half of these matings occurring before 19:00. Therefore, the delay in breeding site arrival time by anthropogenic noise may cause males to miss this important “window of opportunity” when mate attraction is most likely and predation is least likely. Anthropogenic noise has also led to a decrease in the overall time spent chorusing in several anuran species, possibly reducing male reproductive success (Kaiser et al. 2011). However, shifts in breeding activity due to anthropogenic sensory pollutants may, in some cases, confer fitness benefits. In several dawn-chorusing bird species, earlier singing in response to ALAN has also been associated with increased male mating success by increasing extra pair copulations (Kempenaers et al. 2010). Ultimately, subsequent research on the effects of these sensory pollutants on receivers (including potential mates and predators) are necessary to fully understand the fitness implications of changes in male breeding behavior to anthropogenic noise and light.

In addition to the temporal shifts in male breeding behavior, we also found that sensory pollutants can influence male calling effort. Specifically, we found that exposure to ALAN increased calling in both the isolated and the multisensory pollution treatment, whereas noise had no effect. Previous work has shown that urban environments lead to increased calling activity, with higher call rates and complexity (Halfwerk et al. 2019). Although we did not find differences in other signaling traits, the results on calling effort suggest that a single factor, ALAN, may contribute to the differences found between urban and forest males. In our study calling activity was measured only when a single male was calling. The calling behavior of this species is, however, highly socially modulated (Bernal et al. 2009). In chorus settings, with multiple males calling simultaneously, increased calling effort would likely lead to competing males calling simultaneously, thereby increasing call complexity and call rate. Such positive feedback loops due to male–male interactions would likely amplify the effects of artificial light, but future experiments where breeding sites facilitate larger male aggregations, e.g., by increasing breeding site size or lengthening exposure times, are needed to validate this assumption.

Surprisingly, we found no effect of noise on signaling traits, including call rate or call complexity. Responses of anuran species to anthropogenic noise is highly varied, with call rates of species either increasing, decreasing, or remaining unaffected (Kaiser et al. 2011; Caorsi et al. 2017). In the túngara frog, previous work has suggested that this species increases both call rate and complexity in response to low-frequency filtered noise (Halfwerk et al. 2016). The discrepancies between these findings are likely due to: 1) lower noise amplitude in this study; 2) lower spectral overlap between túngara frog calls and our noise playback; and 3) the recording environment (e.g., in a laboratory setting versus in the field). Additionally, although we did not see an effect of noise on any calling characteristics measured, noise may still affect features of these acoustic sexual signals. The characteristics measured in this study were primarily structural in nature, such as call rate and call complexity. However, we did not measure spectral signal traits, which may be altered in response to anthropogenic noise, including call frequency (Wood and Yezerinac 2006; Halfwerk and Slabbekoorn 2009; Parris et al. 2009) and call amplitude (Nemeth et al. 2013; Halfwerk et al. 2016).

In the current study, we document multiple influences of ALAN and anthropogenic noise on frog breeding behavior even though exposure to each sensory pollutant treatment only lasted for a single night. In the context of urbanization, however, persistent populations are exposed to these sensory pollutants for much longer periods of time and across other ecological contexts (e.g., foraging). Longer-term field studies will therefore provide not only a more ecologically relevant scenario, but may also reveal differences between short-term and long-term exposures that may be caused by different behavioral, physiological, or ecological mechanisms. Our results do, however, suggest that urban sensory pollutants can immediately alter a variety of breeding behaviors, which may in turn negatively impact animals through a mismatch in optimal timing of signaling, although this could be negated by potential positive effects of increased signaling effort. Understanding the overall fitness consequences of multisensory pollution in relation to breeding behavior remains a relatively open field, and one that must be further studied in order to properly grasp the evolutionary and ecological ramifications of urbanization.

## Notes

We thank the research and support staff at the Smithsonian Tropical Research Institute, and particularly all staff on Barro Colorado Island, for facilitating data collection. We are grateful to Janine Mariën for scoring audio recordings, and to the Vrije Universiteit workshop for their help designing and building the playback equipment used in this study.

Data Availability: Analyses reported in this article can be reproduced using the data provided by Cronin (2022).
